# Biochemical Mechanisms beyond Glycosphingolipid Accumulation in Fabry Disease: Might They Provide Additional Therapeutic Treatments?

**DOI:** 10.3390/jcm12052063

**Published:** 2023-03-06

**Authors:** Giovanni Bertoldi, Ilaria Caputo, Giulia Driussi, Lucia Federica Stefanelli, Valentina Di Vico, Gianni Carraro, Federico Nalesso, Lorenzo A. Calò

**Affiliations:** Nephrology, Dialysis and Transplantation Unit, Department of Medicine, University of Padova, 35128 Padova, Italy

**Keywords:** Fabry disease, oxidative stress, mitochondria dysfunction, impaired autophagy, antioxidant treatment

## Abstract

Fabry disease is a rare X-linked disease characterized by deficient expression and activity of alpha-galactosidase A (α-GalA) with consequent lysosomal accumulation of glycosphingolipid in various organs. Currently, enzyme replacement therapy is the cornerstone of the treatment of all Fabry patients, although in the long-term it fails to completely halt the disease’s progression. This suggests on one hand that the adverse outcomes cannot be justified only by the lysosomal accumulation of glycosphingolipids and on the other that additional therapies targeted at specific secondary mechanisms might contribute to halt the progression of cardiac, cerebrovascular, and renal disease that occur in Fabry patients. Several studies reported how secondary biochemical processes beyond Gb3 and lyso-Gb3 accumulation—such as oxidative stress, compromised energy metabolism, altered membrane lipid, disturbed cellular trafficking, and impaired autophagy—might exacerbate Fabry disease adverse outcomes. This review aims to summarize the current knowledge of these pathogenetic intracellular mechanisms in Fabry disease, which might suggest novel additional strategies for its treatment.

## 1. Introduction

Fabry Disease (FD, OMIM 301500), the second-most prevalent lysosomal storage disorder (LSD) after Gaucher disease [1], is a monogenic inherited X-linked disease caused by mutations in the alpha galactosidase (GLA) gene, which encodes for the lysosomal enzyme alpha-galactosidase A (α-GalA, EC 3.2.1.22). So far, more than 900 mutations have been listed in the Human Gene Mutation Database (http://www.hgmd.cf.ac.uk, Institute of Medical Genetics in Cardiff, accessed on 12 February 2023), with a high percentage of missense/nonsense mutations and—with less frequency—other types of mutations, such as small deletions, splicing, and small insertions.

FD is pan-ethnic, but due to its rarity and complex clinical picture, a real determination of its frequency is still under scrutiny. The most widely reported incidence, in fact, ranges between 1:40,000 and 1:117,000 in the general population [2,3], but the true prevalence may be largely underestimated. In this regard, two different studies upon newborn screenings found an unexpectedly high prevalence of the disease [4,5].

**Figure 1:**
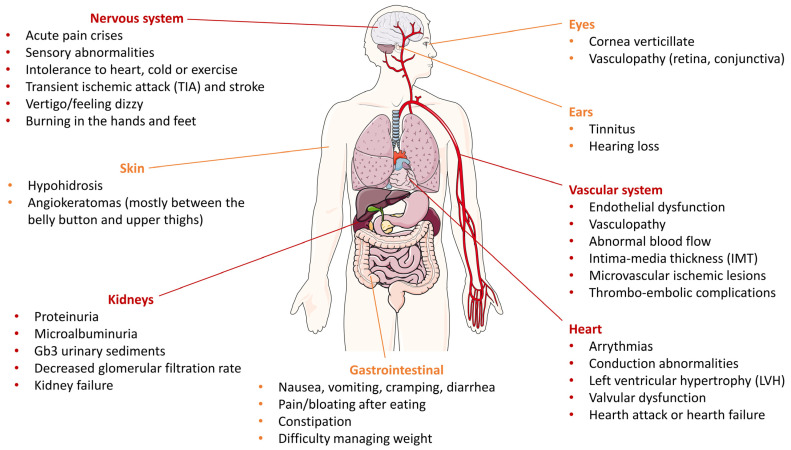
Multiorgan involvement in a classic Fabry patient. Orange markings refer to organs related to severe but not life-threatening symptoms; in red, organs (cerebrovascular system, kidneys, and cardiovascular system) whose involvement is the major cause of death in Fabry patients. The figure was generated using Servier Medical Art, provided by Servier, licensed under a Creative Commons Attribution 3.0 Unported License.

α-GalA takes part in the catabolism of macromolecules, especially glycosphingolipids such as globotriaosylceramide (Gb3), catalyzing the hydrolytic removal of the terminal portion of galactose, leading to the final production of lactosylceramide and free galactose [6]. The resulting reduced or altered enzymatic activity causes a progressive accumulation of Gb3 and derivatives within lysosomes. In common with several LSDs, in FD there is well-documented evidence about a build-up of deacetylated glycosphingolipids [7]. Plasma levels of lyso-Gb3, the deacetylated form of Gb3, were found to be extensively high in both males and females with classical FD compared to healthy controls [8]. Therefore, lyso-Gb3 has become a hallmark diagnostic biomarker of FD [9]. As a result, the progressive accumulation of Gb3 and lyso-Gb3 in plasma and lysosomes of a wide range of cells, including capillary endothelial cells, renal cells (podocytes, tubular cells, glomerular endothelial, and mesangial and interstitial cells), cardiac cells (cardiomyocytes and fibroblasts), and nerve cells, leads to the onset of a multisystemic storage disorder [10] (Figure 1). The wide spectrum of symptoms is also the result of a very high phenotype variability—even within the same family, in which disease manifestations may vary—further complicating the correlation between genotype and phenotype [10,11].

Generally, FD phenotypes can be classified as classic, with typical multi-organ involvement, and later-onset, predominantly with worse cardiac outcomes, with frequencies of up to 1 in 22,570 males and 1 in 1390 males, respectively [12]. Overall, the primary causes of death are due to renal, cardiovascular, and cerebrovascular implications, with a life expectancy of 58.2 years for males and 74.7 years for affected women [13,14].

The main cardiac manifestations are arrhythmias, conduction abnormalities, left ventricular hypertrophy (LVH), valvular dysfunction, myocardial infarction, heart failure and advanced cardiomyopathy [15]. However, it has been found that compared to overall cardiac hypertrophy, the contribution from lipid accumulation is only 1–2% of the cardiac mass, suggesting that lipid storage activates secondary signaling pathways that lead to hypertrophy, apoptosis, necrosis, and fibrosis [16].

Cardiac and renal implications are closely linked. LVH, diastolic dysfunction and arrhythmia induce a reduction in renal perfusion and cause renal venous congestion. On the other hand, reduced glomerular filtration rate leads to retention of water and salts, promoting a predisposition to electrical conduction dysfunction in the heart. FD affects the kidneys in almost all male and in many female patients with the classic phenotype, evolving into end-stage renal disease (ESRD) and early death [17]. The first renal symptoms occur around 4–16 years of age with proteinuria and the presence of Gb3 urinary sediments. In adulthood, microalbuminuria leads to progressive renal dysfunction, worsening of proteinuria, decreased glomerular filtration rate, azotemia, and onset of fibrosis, sclerosis, and tubular atrophy [18]. Hemodynamic derangements likely result in the activation of the renin–angiotensin–aldosterone-system (RAAS) and the release of mediators that induce systemic vasoconstriction. In fact, the resulting elevation of blood pressure is as an important risk factor in the progression of FD [19].

It is noteworthy that another hallmark of the disease is severe neuropathic pain, which occurs in patients as peripheral neuropathy, acute pain crises, sensory abnormalities, hypersensitivity to mechanical stimuli, gastrointestinal pain, and heat and cold intolerance that drastically impacts their quality of life and everyday function [20,21].

Overall, the multiorgan complications of FD are worsened by a chronic inflammatory response triggered by Gb3 and lyso-Gb3 accumulation, which leads to increased pro-inflammatory and pro-fibrotic cytokines, leukocyte perturbation, and overexpression of immune response [22]. At the renal level, lyso-Gb3 was shown to induce an inflammatory and fibrogenic response in terms of increased expression of transforming growth factor β1 (TGF-β1), production of CD47 and induction of epithelial-to-mesenchymal transition [23,24,25]. The inflammatory phenotype is also critical for the development of FD cardiomyopathy, as described by the association of pro-inflammatory biomarkers such as interleukin (IL)-6 and monocyte chemoattractant protein-1 (MCP-1) with specific cardiac complications [26]. Moreover, endomyocardial biopsies from Fabry patients show infiltration of inflammatory macrophages, suggesting a key role of these cells in FD myocardial injury [27]. This wide and complex variety of phenotypes leads to an overlap of symptoms with more common diseases that explains why FD patients are often unrecognized and why diagnosis is delayed for many years (13.7 ± 12.9 for males, 16.3 ± 14.7 for females) [13]. A retrospective study over 58 FD from several internal medicine departments in France revealed that in fact FD is often not the first diagnosis [28].

Currently, the available therapies for Fabry patients are the enzyme replacement therapy (ERT) and the chaperone therapy. ERT consist of the intravenous administration of a recombinant form of α-GalA in order to replace the impaired enzymatic activity and reduce intracellular storage of Gb3 and lyso-Gb3. The oral chaperone—an iminosugar called migalastat chemically similar to galactose, the catalytic product of α-GalA—reversibly binds to the catalytic site of the enzyme, promoting the correct refolding of the protein in the endoplasmic reticulum, its maturation, and trafficking to the lysosomes [29]. However, since the pharmacodynamic of the drug requires interactions with specific amino acid residues of the active site, only certain FD-related GLA mutations are amenable to treatment with migalastat. Worth mentioning are new therapeutic strategies currently under investigation in clinical trials, such as: novel ERT formulations such as a pegylated α-GalA, which renders ERT less immunogenic; substrate reduction therapy (SRT), which acts upstream on Gb3 and lyso-Gb3 synthesis; promising gene therapy in terms of mRNA therapy and ex vivo and in vivo gene therapy [30].

Although short-term studies on the effect of ERT have reported a reduction in left ventricular mass [31] as well as a decrease in neuropathic pain and an overall improvement in cardiac and renal outcomes [32], long-term studies showed that ERT does not alter the natural course of cardiac, cerebrovascular, and renal disease [33,34]. Interestingly, the comparison of data from the literature related to the annualized changes from baseline in the estimated glomerular filtration rate (eGFR) and left ventricular mass index (LVMi) in male patients either untreated or treated with ERT or migalastat showed an incomplete recovery of renal and cardiac functions [35]. This suggests that Gb3 storage alone is unable to explain the observed cardiac and renal outcomes and that secondary biochemical processes might be implicated in FD, potentially suggesting new therapeutic strategies. Hence, the expert recommendation is to combine the available therapies with supportive interventions to clinically manage renal, cardiac, neurological, and other complications of FD-induced chronic tissue injury [11].

In this review, we will summarize and discuss the current knowledge on specific secondary biochemical mechanisms that might concur to exacerbate FD adverse outcomes both in the short and long terms, with a final focus on how a modulation of this mechanism could lead to new therapeutic strategies.

## 2. Altered Pathways in Fabry Disease

**Figure 2:**
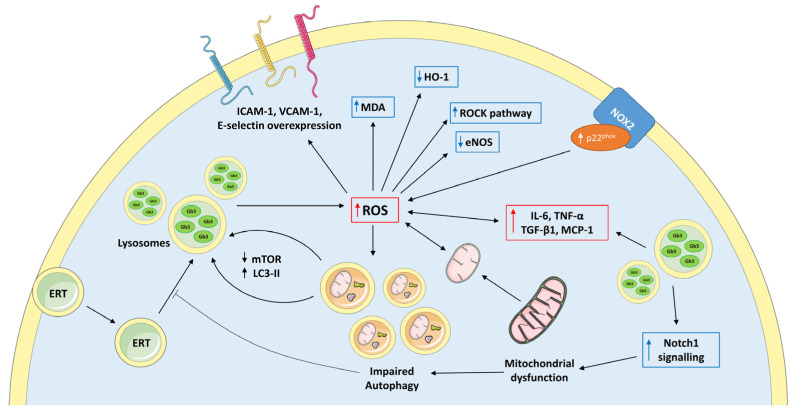
Overview of the main intertwined secondary intracellular mechanism involved in Fabry pathophysiology. The oxidative imbalance and the pro-inflammatory state triggered by Gb3 accumulation, along with overactivation of NOX2 and mitochondria dysfunction, led to impaired autophagy and altered intracellular trafficking, possibly providing a mechanistic explanation of the inability of ERT to halt disease progression. ERT, enzyme replacement therapy; ICAM-1, intercellular adhesion molecule-1; VCAM-1, vascular cell adhesion molecule-1; mTOR, mammalian target of rapamycin; LC3-II, microtubule-associated protein light-chain 3; MDA, malondialdehyde; HO-1, heme oxygenase 1; ROCK, rho kinase; eNOS, endothelial nitric oxide synthase; ROS, reactive oxygen species; NOX2, NADPH oxidase 2; IL-6, interleukin-6; TNF-α, tumor necrosis factor alpha; TGF-β1, transforming growth factor β1; MCP-1, monocyte chemoattractant protein-1. The figure was partly generated using Servier Medical Art, provided by Servier, licensed under a Creative Commons Attribution 3.0 Unported License.

The evidence that the available pharmacological treatments fail to completely resolve FD adverse outcomes, mainly at cardiac and renal level, raised the focus on secondary biochemical processes beyond the accumulation of Gb3 and derivatives that might be involved in FD pathogenesis. These may include oxidative stress (OxSt), compromised energy metabolism, impaired autophagy, and disturbed cellular trafficking [36,37,38] (Figure 2).

### 2.1. Oxidative Stress in Fabry Disease

OxSt occurs when the physiologically generated oxidizing species overwhelm the endogenous antioxidant defenses, resulting in the disruption of the intracellular redox homeostasis [39]. OxSt plays, in fact, a key role in the onset and progression of endothelial dysfunction, atherosclerosis, inflammatory disease, and cardiovascular–renal remodeling [40,41].

In FD, the presence of OxSt was firstly assumed in terms of elevated markers of inflammation and oxidative damage. Shen et al. documented a correlation between Gb3 and increased reactive oxygen species (ROS) production in cultured vascular endothelial cells along with overexpression of intercellular adhesion molecule-1 (ICAM-1), vascular cell adhesion molecule-1 (VCAM-1), and E-selectin [42]. Moreover, endothelial cells incubated with plasma from FD patients revealed higher ROS generation compared to treatment with plasma from healthy subjects [42].

OxSt also causes damage at nuclear and genomic levels, which results in free radicals damaging DNA and inducing changes in gene expression. Levels of 8-hydroxy-2-dehydroguanosine (8-OHdG), one of the most abundant by-products of DNA oxidation and therefore a non-invasive biomarker of OxSt [43], were found elevated both in the serum [44] and in the myocardial tissue [45] of FD patients with cardiomyopathy, and damage at the DNA level does not seem to be fully restored by endogenous repair mechanisms in FD [46].

The tight association of OxSt with NO dysregulation was also explored in FD. It has been suggested that Gb3 buildup in the endothelium is able to dysregulate the activity of the vasoprotective endothelial nitric oxide synthase (eNOS) while increasing the expression of the inducible NOS (iNOS) [47,48]; iNOS produces larger amounts of NO compared to eNOS, which can easily react with other free radicals, producing reactive nitrogen species (RNS) such as peroxynitrite and spreading the oxidative damage to surrounding macromolecules [49]. In addition, α-GalA knockdown human endothelial cell line showed dramatically enhanced production of 3-Nytrotyrsine (3NT), a marker of nitroxidative damage of proteins, in addition to Gb3 accumulation and reduced eNOS activity [47]. Excesses of 3NT were also found in the dermal and cerebral vessels of FD patients [50] and in the plaque of apolipoprotein-E-deficient mice with α-GalA deficiency and accelerated atherosclerosis [51]. Moreover, tetrahydrobiopterin—an essential cofactor for the normal enzymatic function of NOS—was decreased in the heart and kidney of an animal model of FD and inversely correlated with Gb3 levels in animal tissue and cultured patient cells [52].

Different comparative studies showed activation of OxSt-related pathways in FD compared to healthy subjects. Biancini et al. showed that FD patients exhibit decreased levels of antioxidant defenses, such as glutathione (GSH) and GSH peroxidase (GPX); higher plasma levels of malondialdehyde (MDA), protein carbonyl groups and di-tyrosine in urine together with increased proinflammatory cytokines IL-6 and tumor necrosis factor alpha (TNF-α) [53]. In addition, urinary Gb3 levels were positively correlated with IL-6, carbonyl groups, and MDA plasma levels, suggesting a link between proinflammatory and prooxidant conditions, both likely induced by Gb3 accumulation [53]. This impaired oxidative profile of Fabry patients was further demonstrated by our group, particularly linking the overactive OxSt with the induction of cardiovascular–renal remodeling, which is distinctive of FD [54]. We have documented, in fact, an overactivation of the mechanism directly involved in ROS production in terms of increased expression of p22^phox^ (subunit of the NADPH oxidase, NOX), a downregulation of heme oxygenase 1 (HO-1), endogenous antioxidant defense, and increased levels of MDA, a marker of lipid peroxidation [54]. It is of note that bilirubin, a potent antioxidant by-product of HO-1 catabolism, was found to be decreased in Fabry patients compared to controls along with reduced total antioxidant status (TAS) [55]. Furthermore, we have shown that the Rho kinase (ROCK) pathway [56,57] is activated in Fabry patients in terms of increased phosphorylation of its target MYPT-1, suggesting that ROCK activation also plays a crucial role in the oxidative signaling and cardiovascular–renal remodeling of Fabry patients [54]. Therefore, it is reasonable to state that these pro-oxidant molecules may represent early biomarkers of the disease, as was already suggested in FD with reference to the advanced oxidation protein products (AOPP), ferric reducing antioxidant power (FRAP), and thiolic groups [58].

These data further support the hypothesis that OxSt plays an important role in the pathogenesis of the disease with reference to the vascular damage and the cardiovascular-renal remodeling that occur in FD. A better understanding of the specific molecular signaling responses to OxSt in FD could suggest new treatment strategies in order to reduce the high morbidity of these patients.

### 2.2. Mithocondrial Dysfunction in Fabry Disease

Mitochondria are known as the cellular energy powerhouse in addition to being an important source of ROS [59]. Mitochondrial ROS (mtROS) are produced as side-products of the oxidative phosphorylation process [60], and a perturbation of the mitochondrial redox homeostasis and the resulting excessive accumulation of mtROS have been linked to the development of several diseases, including cancer, pulmonary and cardiovascular disease, neurodegenerative disorders and diabetes [61].

Mitochondria disfunction has emerged as an harmful factor in the pathophysiology of LSDs, including Gaucher’s disease, Niemann–Pick disease and mucopolysaccharidosis [62]; a dysfunctional mitochondria may in fact impact lysosomal function via generation of ROS as well as depriving the lysosome of ATP, which is required by the V-ATPase proton pump to maintain the acidity of the vacuoles [63]. Insights into a role of mitochondria dysfunction in FD were provided by Lücke and colleagues that documented a reduced activity of mitochondrial respiratory chain (MRC) complexes in skin fibroblasts from FD patients in terms of decreased activity of MRC complex II, IV, and V compared to controls [64]. Impaired oxidative phosphorylation and mitochondrial metabolism as indicated by decreased production of high energy phosphate molecules (e.g., ATP and creatine phosphate) were detected in the hearts of FD subjects [37,65], and downregulation of mitochondrial endonuclease G—critical for cardiac mitochondrial function [66]—was observed in Fabry cardiomyocytes [67]. At the renal level, disturbed mitochondrial structure, metabolism, and turnover were documented in renal tubular epithelial cells from male FD patients [68]. Furthermore, lyso-Gb3 accumulation in podocytes determined the upregulation of the pro-inflammatory and profibrotic Notch1 signaling pathway [69], whose overactivation affects the mitochondrial proteome and impairs mitochondrial metabolism [70]. Additional evidence on the link between OxSt and mitochondrial dysregulation in FD comes from Tseng et al. [71] that reported a downregulation of the protein expression of the superoxide dismutase 2 (SOD2), a mitochondrial antioxidant, in FD-specific human-induced pluripotent stem cells, which was associated with increased ROS generation and enhanced intracellular Gb3 buildup.

Mitochondria are uniquely characterized by their own DNA (mtDNA), a closed-circle double-stranded DNA without histones, which encodes for 37 genes, including 13 components of the mitochondrial electron transport chain [72]. Interestingly, Simoncini et al. investigated whether specific genetic polymorphism in the mitochondrial genome of a cohort of 77 Fabry patients could be accountable for specific FD phenotypes [73]. They showed that certain haploid groups were more prevalent in patients, although there was no observed correlation with gender, age of onset, or organ involvement [73].

The mitochondrial genome is also able to produce specific microRNAs (miRNAs), called mitomiRs, involved in the regulation of mitochondria protein expression and function of mitochondria-related biological processes such as energy metabolism, mitochondrial OxSt, and apoptosis [74]. Recently, a mitomiR dysregulation was unveiled in Fabry patients, shedding light on mitochondria miRNA as critical players in contributing to their aberrant mitochondrial homeostasis and pointing to mitomiRs as a novel class of biomarkers of FD [75].

### 2.3. Impaired Autophagy in Fabry Disease

Lysosomes are critically involved in the maintenance of cellular homeostasis, driving the degradation and recycling of metabolic wastes, dysfunctional organelles such as mitochondria (i.e., mitophagy [76]), or large cytosolic molecules, especially in response to environmental stress. This process takes place via the autophagy–lysosome pathway (ALP), a process that seizes cytoplasmic cargos into double-membraned vesicles called autophagosomes and direct them toward lysosomes [77]. Since autophagy plays a key role in the clearance of lysosomal substrates [78], increasing evidence suggests a key role of autophagic (dys)function in FD pathophysiology. In fact, several studies reported an autophagic dysregulation in different models of FD. Chévrier et al. firstly documented an upregulation of the autophagic marker microtubule-associated protein light-chain 3 (LC3-II) in human Fabry skin fibroblasts and in kidney biopsies along with accumulation of autophagic vacuoles in FD renal cells, assuming a disturbance of the autophagic pathway [79]. Alteration of autophagic pathways was further confirmed in α-GalA deficient podocytes that, in addition to marked upregulation of LC3-II levels, showed a decreased activity of the mammalian target of rapamycin (mTOR) kinase, a negative regulator of autophagic vesicle formation, suggesting that the overactive autophagy, beyond Gb3 accumulation, may be an additional contributor to podocyte damage and glomerular injury occurring in Fabry patients [80]. Moreover, autophagy was recently addressed as a key mechanism triggering renal tubulointerstitial fibrosis in an animal model of FD [81]. The role of autophagy disorders in FD was also assessed in the context of neurological [82] and corneal [83] complications.

In LSDs, the lysosomal trapping of sphingolipids within lysosomes and the altered lipid recycling and trafficking has a direct effect on the lipid composition and the biophysical properties of plasma and intracellular membranes [37,84]. In FD, changes in the lipid composition of membranes were assessed in patients’ fibroblasts [85], in the inner mitochondrial membrane [86], and in specialized intracellular membrane domains termed lipid rafts [87]. Consequently, changes in the lipid composition may alter the stoichiometry and functionality of their protein components (e.g., ion channels [88,89]), further perturbing the signal transduction.

This disturbed intracellular environment might also affect ERT efficacy since the therapeutic effect relays on the proper intracellular uptake of the infused enzyme via the endolysosomal pathway. After the binding of the administered enzyme with a membrane receptor—such mannose-6-phosphate receptor (M6PR), megalin, or sortilin [90]—an essential component in the trafficking is the generation and maintenance of a proper low endolysosomal pH gradient, which enables dissociation between the receptors and their ligands, the recycling of receptors back to the apical membrane, and the progression of the vesicles toward lysosomes [91]. A key protein involved in the vesicular acidification is the chloride channel Cl^−^/H^+^ antiporter ClC-5, mainly located in the early endosomes [91]. In this regard, in renal proximal tubule cells, a loss of ClC-5 was associated with trafficking defect along with lysosomal dysfunction, OxSt, and dedifferentiation [92]. Moreover, a downregulation of ClC-5—along with cubilin and megalin, two endocytic receptors responsible for the reabsorption of the vast majority of proteins filtered in the glomeruli—was cytologically assessed in renal biopsies of two cases of FD [93]. Therefore, it is reasonable that ClC-5 downregulation and the following impaired intracellular trafficking might affect and limit the therapeutic effect of ERT towards the natural progression of the disease.

Finally, the above-described endosomal–lysosomal abnormalities have been recently associated with the likely protection from COVID-19 that Fabry patients seem to exhibit, in view of the unfavorable host cellular environment for SARS-CoV-2 infection and propagation, that does not seem to be affected by ERT treatment [94]. However, a molecular mechanistic explanation of this naturally occurring protection is still unknown.

## 3. Potential Additional Therapeutic Strategies in Fabry Disease

Combined, these observations suggest that targeting specific pathways related with OxSt, mitochondria dysfunction, and aberrant autophagy can represent a potential additional therapy for the treatment of Fabry patients. However, relatively few preclinical or clinical trials have explored the effect of a restored oxidative balance, mitochondria, or autophagy function in FD or more generally in LSDs.

Upregulation of mTOR signaling was suggested as a novel strategy to improve impaired autophagy and restore a functional autophagosome–lysosome fusion process [95]. L-arginine, an amino acid easily taken as a natural dietary supplement, acts as an activator of the mTOR complex 1 (mTORC1) by suppressing the lysosomal localization of the tuberous sclerosis complex (TSC), a GTPase-activating protein (GAP) that converts active GTP-bound Ras homolog enriched in brain (Rheb) to inactive GDP-bound form, inhibiting mTORC1 activity [96]. In the context of LSDs, a therapeutic modulation of mTOR via L-arginine was assessed in a mouse model of Pompe disease [97]. In this study, in vivo and in vitro L-arginine supplementation was able to reverse the aberrant mTOR signaling, resulting in a significant removal of autophagic buildup [97]. Recently, phospholipid–polyethyleneglycol-capped ceria-zirconia nanoparticles (PEG-CZNPs) were shown to restore a physiological autophagic flux, increasing mTOR pathways and leading to a reduction of Gb3 accumulation, decreasing OxSt and attenuating kidney injury in both cellular and animal models of FD [98].

These data shed light on the critical role that a specific nutritional approach can have in the management of FD, considering that specific dietary interventions are recommended in renal involvement and that some dietary approaches could improve gastrointestinal symptoms of Fabry patients [99]. In most FD patients, gastrointestinal symptoms mimic irritable bowel syndrome (IBS), for which the main nutritional recommendations include reducing intake of caffeine, alcohol, insoluble fiber, fat, spices, and spicy foods. Consequently, some specific dietary patterns and bioactive compounds present in foods may interfere with specific defective intracellular pathways that induce tissue-damaging effects.

With reference to the antioxidant approaches, very few studies in the literature tested an antioxidant treatment in FD. The first—published in 1987, before ERT approval—investigated the effects of vitamin E and ticlopidine on platelet aggregation in order to treat or prevent thromboembolism, frequently observed in Fabry patients [100]. After 7 days of treatment, a moderate inhibitory aggregation effect was seen after vitamin E administration, while platelet aggregation was completely improved by ticlopidine. In another study, ascorbate, a potent antioxidant, was found to decrease cerebral hyperperfusion in Fabry patients under ERT treatment [101]. Recently, Kim et al. showed in an in vitro disease model of renal FD that treatment with the antioxidant GSH reduced OxSt and attenuated the structural alterations of the GLA-mutant kidney organoids compared to wild-type (WT) kidney organoids [102]. The same relief from OxSt and restoration of the deformed cellular structure was obtained by treating these organoids with ERT via recombinant human α-Gal A (rhα-GLA) at different concentrations [102]. Interestingly, this study documented in vitro a potential antioxidant effect held by ERT itself although in terms of attenuation and not restoration, as the levels of oxidative markers remained higher than WT kidney organoids [102]. However, the combined effect of ERT and GSH was not tested. Therefore, no evidence can be drawn from this study about the efficacy of an antioxidant additive treatment in FD. Regarding the effect of ERT on OxSt, Biancini et al. compared the differences in the oxidative profile between a group of Fabry patients not receiving ERT, a group of Fabry patients under ERT, and healthy subjects [103]. What has been observed is that Fabry patients on ERT presented GSH metabolism similar to controls, although lipid peroxidation and urinary levels of NO• equivalents remained higher in treated and untreated patients [103]. In a preliminary study, we provided a mechanistic rationale to the relevance of anti-inflammatory/antioxidative dietary pattern in addition to ERT to contribute to slowing disease progression in FD [104]. We showed that in addition to an antioxidant effect exerted by ERT itself, the antioxidant treatment with green tea supplementation on top of ERT was able to decrease levels of p22^phox^, MDA and MYPT-1 phosphorylation, a marker of ROCK activity, while increasing the endogenous antioxidant defences in terms of HO-1 expression [104], suggesting that the distinctive oxidative imbalance of Fabry patients should be taken into consideration in the management and treatment of FD.

## 4. Conclusions

FD is characterized by a complex clinical picture with an overlap of symptoms with more common diseases, leading to a delay in diagnosis and initiation of therapy. Significant amelioration occurs upon treatment with ERT, especially if started early. However, this does not always lead to a complete restoration. Therefore, the adverse outcomes cannot be justified only by the accumulation of Gb3 and lyso-Gb3, and additional therapies are needed and should be found to modulate aberrant secondary mechanisms that trigger the progression of the disease.

The discussion of the intracellular processes reported in this paper revealed a network of intertwined mechanisms that exacerbate the complex biochemistry of Fabry patients beyond Gb3 and derivatives accumulation. OxSt is a main trigger of impaired autophagy and mitochondria dysfunction in addition to being a key factor in the pathophysiology of renal injury and cardiovascular–renal remodeling. In addition, altered autophagic flux can further trigger secondary lysosomal deposition via the accumulation of dysfunctional mitochondria, which contribute to increased oxidative damage and lipid droplets.

Evidence has been reported that supports positive outcomes with additive treatments directed towards these mechanisms, which, however, need further specific studies to confirm their efficacy in FD.

## Data Availability

No new data were created or analyzed in this study. Data sharing is not applicable to this article.
